# Three-dimensional brain-on-chip model using human iPSC-derived GABAergic neurons and astrocytes: Butyrylcholinesterase post-treatment for acute malathion exposure

**DOI:** 10.1371/journal.pone.0230335

**Published:** 2020-03-12

**Authors:** Lumei Liu, Youngmi Koo, Teal Russell, Elaine Gay, Yan Li, Yeoheung Yun

**Affiliations:** 1 FIT BEST Laboratory, Department of Chemical, Biological, and Bio Engineering, North Carolina Agricultural and Technical State University, Greensboro, North Carolina, United States of America; 2 Center for Drug Discovery, RTI International, Research Triangle Park, Durham, North Carolina, United States of America; 3 Chemical Engineering, Florida A&M University-Florida State University, Tallahassee, Florida, United States of America; University of North Dakota, UNITED STATES

## Abstract

Organophosphates (OPs) induce acute and chronic neurotoxicity, primarily by inhibiting acetylcholinesterase (AChE) activity as well as by necrosis, and apoptosis. Butyrylcholinesterase (BuChE), an exogenous bioscavenger of OPs, can be used as a treatment for OP exposure. It is prerequisite to develop *in vitro* brain models that can study BuChE post-treatment for acute OP exposure. In this study, we developed a three-dimensional (3D) brain-on-chip platform with human induced pluripotent stem cell (iPSC)-derived neurons and astrocytes to simulate human brain behavior. The platform consists of two compartments: 1) a hydrogel embedded with human iPSC-derived GABAergic neurons and astrocytes and 2) a perfusion channel with dynamic medium flow. The brain tissue constructs were exposed to Malathion (MT) at various concentrations and then treated with BuChE after 20 minutes of MT exposure. Results show that the iPSC-derived neurons and astrocytes directly interacted and formed synapses in the 3D matrix, and that treatment with BuChE improved viability after MT exposure up to a concentration of 10^−3^ M. We conclude that the 3D brain-on-chip platform with human iPSC-derived brain cells is a suitable model to study the neurotoxicity of OP exposure and evaluate therapeutic compounds for treatment.

## Introduction

Organophosphates (OPs) are nerve agents that pose a serious threat to military personnel and civilian populations. OPs cause acute and chronic neurotoxicity mainly by inhibition of acetylcholinesterase (AChE) [1]. However, other mechanism are also reported to link with necrosis, apoptosis, and oxidative stress mediated pathway [2]. Butyrylcholinesterase (BuChE) has been explored as a bioscavenger of OPs, preventing them from reaching their physiological targets [3]. BuChE and AChE have similar active sites [4–6] and are both efficient at catalyzing the breakdown of ACh [7]; however, loss of BuChE activity does not lead to toxicity, as BuChE is not known to perform an essential function *in vivo* [8]. Thus, the use of BuChE as a treatment for OP neurotoxicity has been investigated extensively [6, 9–12]. It is currently the only therapeutic agent effective in providing complete stoichiometric protection against the entire spectrum of OP nerve agents without inducing antagonistic immunological responses [13, 14]. Pre- and post-treatment with BuChE has been shown to protect against the toxic effects of OP exposure in animal models [11, 15–17].

Two-dimensional (2D) *in vitro* models and three-dimensional (3D) animal brain slices have long been used as well-accepted models to study brain cellular responses to biophysical and biochemical stimulation. However, 2D models do not accurately replicate the three-dimensional (3D) cytoarchitecture of the brain, and brain slices require animals sacrifice and are not high-throughput. Furthermore, the current static culturing method of transwell technology has limited ability to mimic complex neuronal tissue. The use of human induced pluripotent stem cells (iPSCs) can provide better results for human-relevant brain modeling for toxicity screening, and diseases modeling [18].

In this paper, we developed 3D brain tissue constructs using human induced pluripotent stem cell (iPSC)-derived GABAergic neurons and astrocytes embedded in a 3D matrix with dynamic medium flow. This platform allows for neuron-astrocyte interactions that further enhance neuronal function and *in vivo*-like signatures. First, different ratios of iPSC-derived GABAergic neurons and astrocytes were co-cultured and evaluated for direct interaction via synapse formation. Second, following Malathion (MT) exposure, we evaluated the effects of BuChE treatment in terms of cell viability and cholinesterase (ChE) activity. The results from this *in vitro* platform are correlated with *in vivo* results for validation of the model in toxicity screening and evaluation of therapeutic compounds for treatment of OP exposure.

## Materials and methods

### Cell culture and seeding

Human induced pluripotent stem cell (iPSC)-derived GABAergic neurons and astrocytes (iCell^®^ GABANeurons and Astrocytes) were purchased from FUJIFILM Cellular Dynamics, Inc. (FCDI, Madison, WI). Matrigel^™^ Growth Factor Reduced Membrane Matrix was purchased from Corning^™^ (Corning, NY). The Matrigel was diluted to 5 mg/mL with iCell Neural Complete Maintenance Medium (iCell Neural Base Medium 1 + 2% Neural Supplement A + 1% Penicillin/Streptomycin) on ice. Neurons and astrocytes were mixed in ratios of 4:1 and 1:4 (A1/N4 and A4/N1, respectively) and then embedded in Matrigel. 2 μL of cell-gel matrix was seeded into each gel lane of a 2-Lane OrganoPlate^®^ (MIMETAS, Netherlands) using a repeater pipette (Eppendorf Repeater^®^, E3X, Hauppauge, NY) and then gelled at 37°C and 5% CO2 for 1 h. Next, 20 μL of medium was added to the gel lane, and 50 μL of medium was added to the inlet and outlet of the medium lane (100 μL total) of each well. The plate was incubated at 37°C and 5% CO2 on an interval rocker (MIMETAS, Netherlands) to allow medium perfusion with bi-directional flow. Medium was refreshed every other day.

### Fluorescence microscopy

Cells were fixed with 4% paraformaldehyde (PFA) solution (Affymetrix, Santa Clara, CA, USA) for 15 min, washed twice with phosphate-buffered saline (PBS) for 5 min, permeabilized with Triton X-100 (0.1% in PBS) for 5 min, and blocked with 10% normal donor horse serum in PBS for 1 h at room temperature. Then, cells were incubated with primary antibodies for 1.5 h, washed 3 times with PBS for 3 min, incubated with secondary antibodies for 1 h, and again washed 3 times with PBS for 3 min at room temperature. Neurons were stained with anti-β-tubulin III Alexa Fluor 488 (1:20, BD Biosciences, 560381), and astrocytes were stained with anti-GFAP (1:100, Invitrogen, PA5-18598) and donkey anti-goat Alexa Fluor 647 (1:100, EMD Millipore, AP180SA6). Synapses were stained with anti-Synaptophysin (1:50, Invitrogen, MA5-14532) and donkey anti-rabbit Alexa Fluor 568 (1:50, Invitrogen, A10042), and nuclei were stained with Hoechst (1:2000, Invitrogen, H3570). Fluorescent images were obtained and processed using a two-photon confocal microscope and ZEN software (Carl ZEISS Multiphoton LSM 710, Oberkochen, Germany).

### Malathion exposure and Butyrylcholinesterase treatment

Malathion (MT, Sigma-Aldrich, St. Louis, MO, USA) was diluted in 5% Dimethyl sulfoxide (DMSO) in iCell Neural Complete Maintenance Medium, and then further diluted to concentrations of 10^−1^, 10^−3^, and 10^−5^ M in the same medium. On the 5th day of 3D co-culture, the medium was removed from each well and replaced with 100 μL (50 μL in/outlet) MT-containing medium in replicates of 3 for each MT concentration. After 20 min of MT exposure, one set of replicates was treated with 10 μL (5 μL in/outlet) of 50 μM Butyrylcholinesterase (BuChE, Sigma-Aldrich, St. Louis, MO, USA) in medium. After 24 h, the medium was removed from each well and stored at −20 °C for analysis of ChE activity.

### Cell viability

Cell viability was evaluated using the LIVE/DEAD^®^ Viability/Cytotoxicity Kit for mammalian cells (Invitrogen, Carlsbad, CA). After medium was removed, co-cultures were washed twice with PBS, then incubated for 30 min with 2 μM Calcein AM, 4 μM EthD-1, and 2 μg/mL Hoechst, staining live cells green, dead cells red, and nuclei blue, respectively. Viability was calculated using ImageJ software (US National Institutes of Health, Bethesda, MD) by dividing the number of live cells by the total number of cells and multiplying by 100%.

### Acetylcholinesterase and Butyrylcholinesterase activity assay

The Molecular Probes^™^ Amplex^™^ Acetylcholine/Acetylcholinesterase (AChE) Assay Kit was used to measure AChE and BuChE activities. Briefly, standards and working solutions were prepared according to the manufacturer’s instruction. Samples and standards were then incubated in working solution for 30 min. Fluorescence was read using a CLARIOstar microplate reader (BMG LABTECH, Cary, NC, USA) at room temperature.

### Statistical analysis

Cell viability and AChE activity data (Mean ± Standard deviation) were plotted using Microsoft Excel. The correlation of *in vitro* AChE activity (inhibitor concentration 50%, IC50) and viability (lethal concentration 50%, LC50) data with *in vivo* lethal dose 50% (LD50) data was analyzed using Origin^®^ 2018 (OriginLab, Northampton, MA, USA). Heteroscedastic t-test was conducted on viability using Microsoft Excel for each concentration of MT to compare with and without BuChE. One-way ANOVA and paired t-test were conducted on ChE and AChE activity data using GraphPad Prism version 8.0.0 (GraphPad Software, Inc, San Diego, CA). Statistical differences between compared groups were considered as significant when p-vale < 0.05 (two-tail). P-value is not shown when < 0.0001.

## Results

### Dynamic 3D human brain-on-chip platform

**Fig 1:**
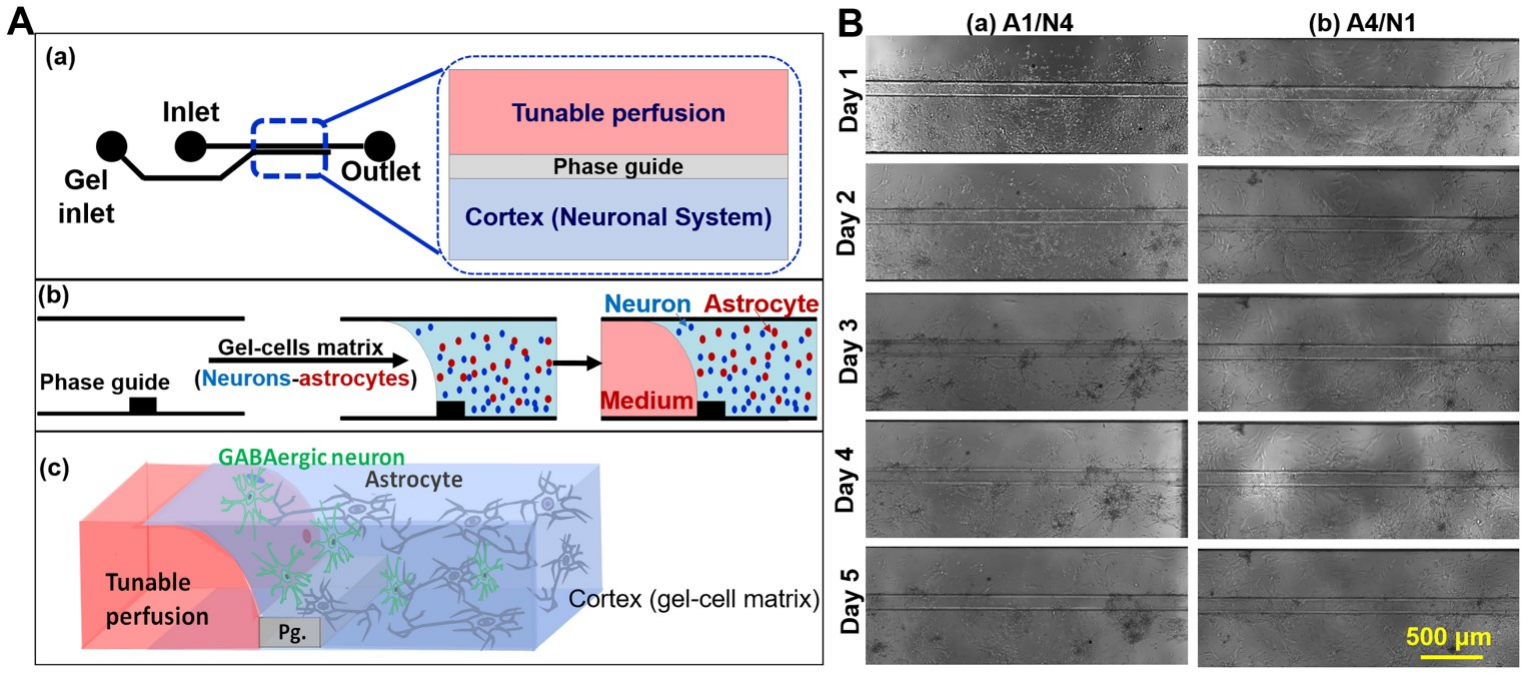
Dynamic 3D brain-on-chip platform using human iPSC-derived GABAergic neurons and astrocytes. (A) Schematic of 3D brain-on-chip platform and brain tissue construct used for high-content MT toxicity and BuChE bioscavenger testing. (B) Bright-field images of brain tissue constructs with different ratios of human iPSC-derived neurons and astrocytes (1:4 (a) and 4:1(b)) taken over the course of 5 days.

The schematic process used to construct the brain-on-chip platform is shown in Fig 1A. We first loaded Matrigel embedded with human iPSC-derived GABAergic neurons and astrocytes into the gel lane, which is separated from the medium lane by a capillary pressure barrier called a phase guide. Following polymerization of the cell-gel matrix, medium was added to each well of gel lane and perfusion lane. Then the plate was kept on a programmable perfusion rocker, which provided shear stress and an average medium flow of 1.5 μL h−1. Fig 1A(c) represents the final brain tissue construct that was successfully co-cultured with GABAergic neurons and astrocytes and ready to be implemented in MT toxicity and BuChE bioscavenger testing. We cultured iPSC-derived GABAergic neurons and astrocytes separately in Matrigel for 5 days in 2D well plate (S1 Fig) and confirmed cell-Matrigel and medium compatibility, in addition to optimizing cell density. We explored two different ratios of astrocytes to neurons, 1:4 (A1/N4) and 4:1 (A4/N1). The ratio A4/N1 was selected according to the ratio of glia to neuron is 10:1 and astrocytes are ~40% population of glia [19]. A1/N4 is the opposite ratio to further explore the effect of different ratio. Fig 1B shows bright-field images of the brain tissue constructs over the course of 5 days.

**Fig 2:**
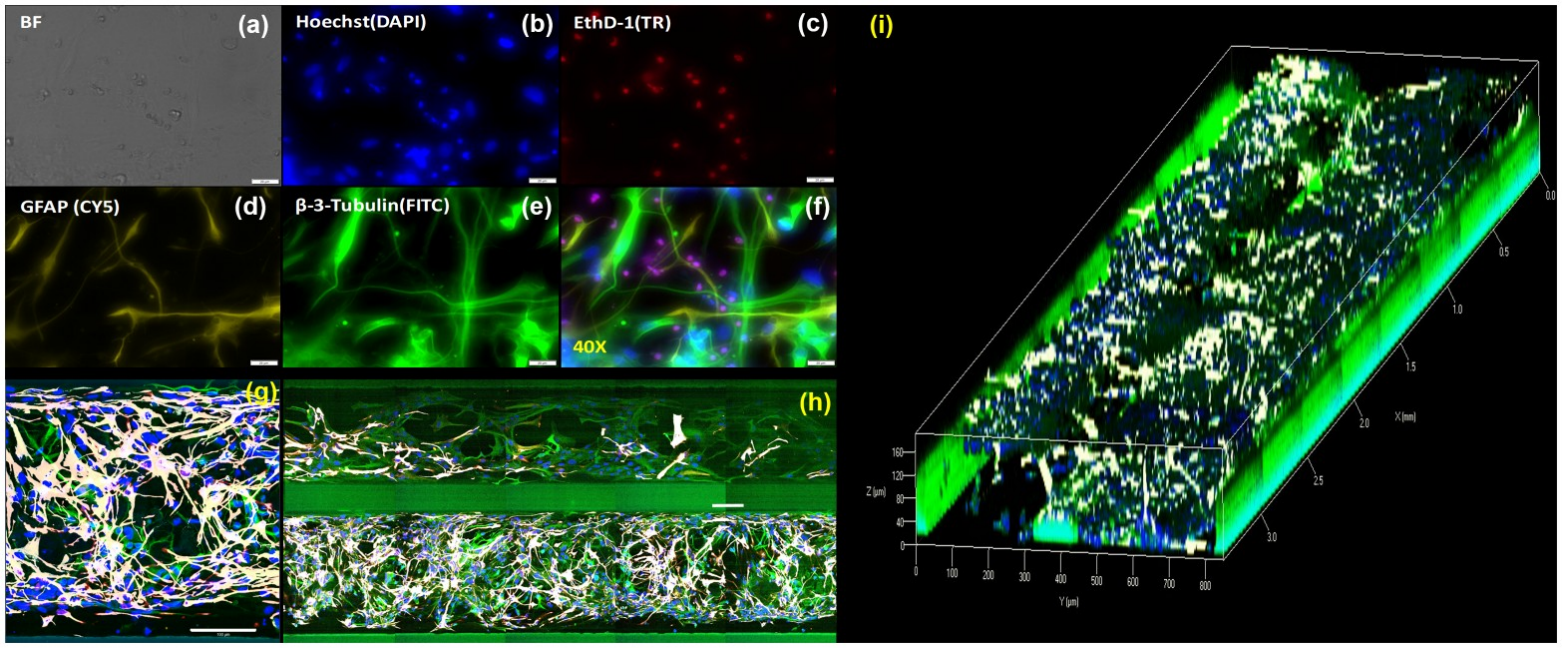
Dynamic 3D brain tissue construct with human iPSC-derived GABAergic neurons and astrocytes. (a) Bright-field image, (b) Hoechst: nuclei, (c) EthD-1: dead cells, (d) GFAP: astrocytes, (e) β-tubulin III: neurons, (f) Overlay image, (g) Top view of neurons (green) and astrocytes (white) in the cell-gel matrix, (h) Top view of brain tissue construct showing cell-gel matrix and perfusion lane, and (i) 3D render of entire brain-on-chip construct. Scale Bar: (a)-(f) = 20 μm, (g)-(h) = 100 μm.

The morphology of co-cultured cells is shown in Fig 2. Bright-field images taken at higher magnifications (Fig 2a) did not give a clear presentation of the cells in 3D matrix. Nuclei (Fig 2b) and dead cells (Fig 2c) were stained blue and red, respectively. Neurons were stained green via β-tubulin III (Fig 2e), while astrocytes were stained via GFAP and are shown in yellow in Fig 2d–2f and white in Fig 2g–2i. High-magnification images (Fig 2a–2f) displayed the interaction between neurons and astrocytes, as well as the distribution of dead cells. Fig 2g–2i show the overall structure of the brain tissue constructs.

**Fig 3:**
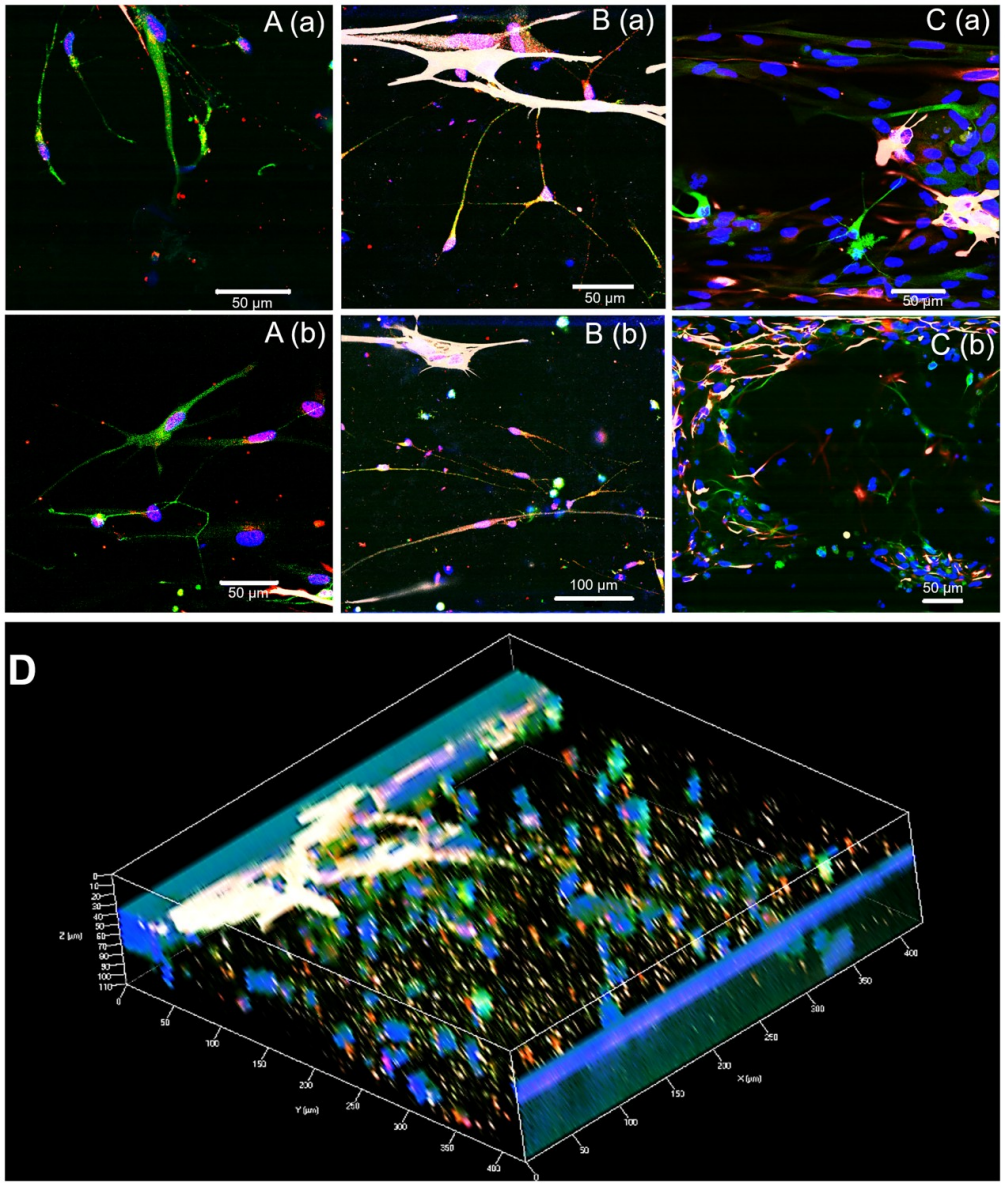
Functional synapses between cells. A. Synapses between neurons (A1/N4); B. Synapses between neurons and astrocytes (A1/N4); C. No synapses in A4/N1 group. D. 3D co-culture with synapses (A1/N4). Red: Synaptophysin for synapses, green: β-tubulin III for neurons, white: GFAP for astrocytes, and blue: Hoechst for nuclei.

Synapses formed between cells were stained via Synaptophysin (red dots in Fig 3). There were clear synapses between neurons (Fig 3A) and neurons and astrocytes (Fig 3B) in the A1/N4 group, but not in A4/N1 group (Fig 3C). The 3D co-culture of synapses (red dots) was shown in Fig 3D for A1/N4 group.

### BuChE post-effect on viability of MT-exposed co-cultures

**Fig 4:**
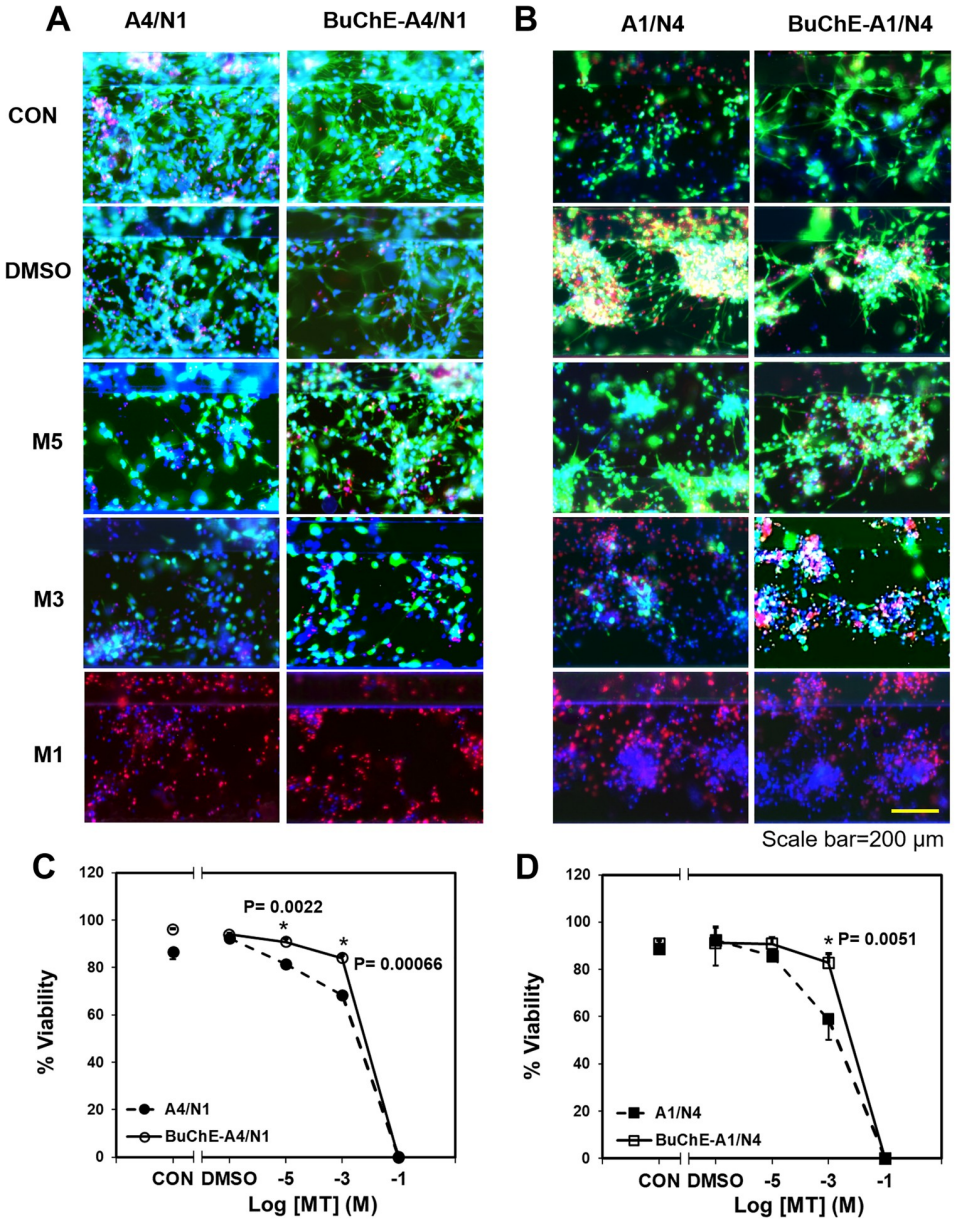
Cell viability following acute MT exposure and BuChE post-treatment in A4/N1 (A, C) and A1/N4 (B, D) groups. Representative live/dead images of 3D co-cultured cells (A. A4/N1, B. A1/N4) exposed to MT with and without BuChE post-treatment. Viability quantification of co-cultured cells A4/N1 (C) and A1/N4 (D). * represents significant difference of cell viability between with and without BuChE (P = 0.0022, 0.00066 for A4/N1 group treated with 10^−5^, 10^−3^ M MT, respectively, P = 0.0051 for A1/N4 group treated with 10^−3^ M MT).

Brain tissue constructs were exposed to MT at concentrations of 10^−1^, 10^−3^, and 10^−5^ M for 24 hours. Viability was analyzed by staining live cells(green) and dead cells(red). In both the A1/N4 and A4/N1 co-cultures, viability decreased with an increase of MT concentration (Fig 4). When BuChE (50 μM) was added 20 minutes after MT exposure, viability increased significantly in constructs exposed to 10^−5^ M and 10^−3^ M MT in the A4/N1 group and 10^−3^ M MT in the A1/N4 group (P = 0.0022, 0.00066, 0.0051 respectively, Heteroscedastic t-test). DMSO (5%) was used as MT solvent, and the results showed that DMSO did not affect cell viability.

### BuChE as MT bioscavenger: AChE and ChE activity

**Fig 5:**
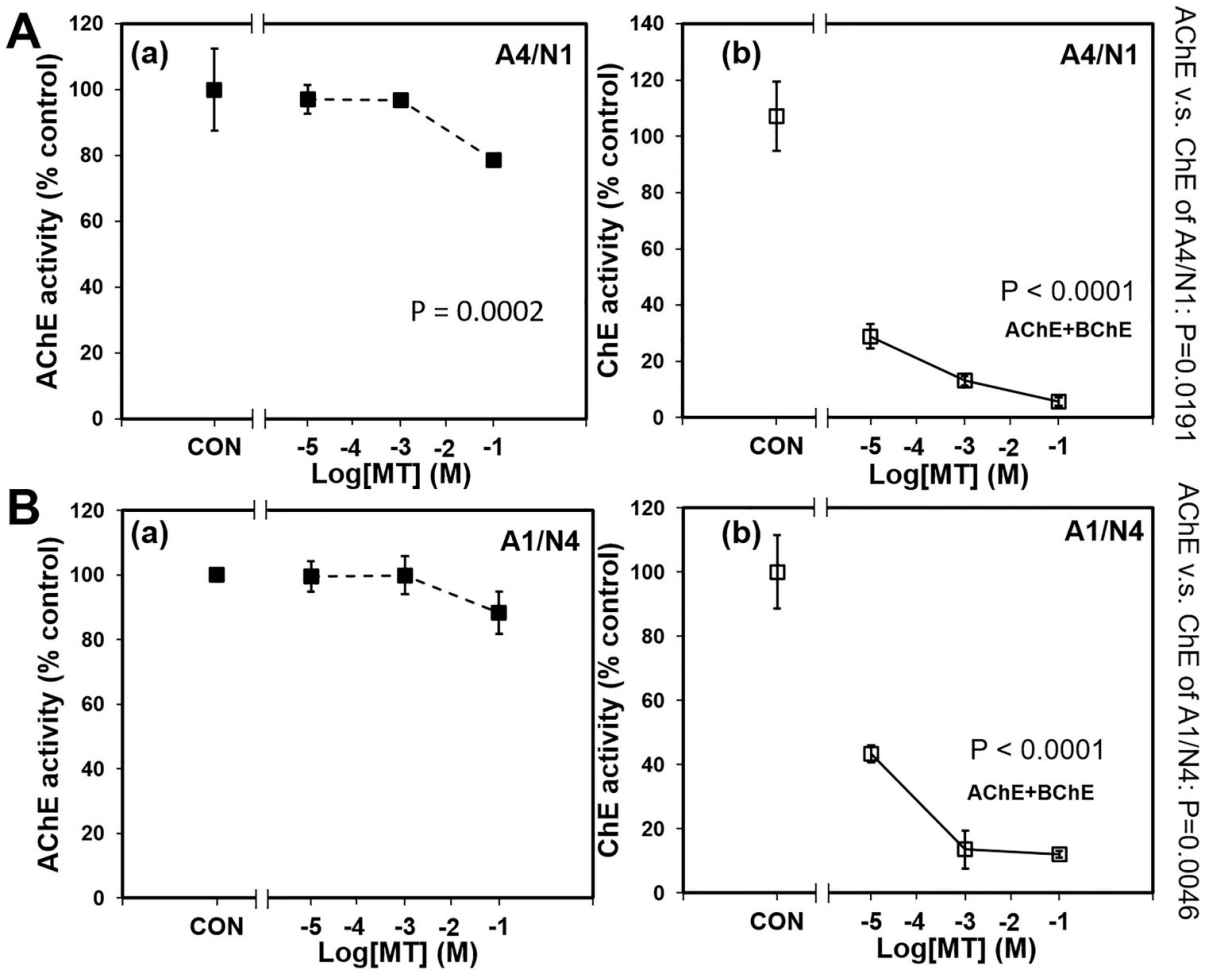
AChE and total ChE activity of brain tissue constructs following acute MT exposure. (A) A4/N1, (B) A1/N4, (a) AChE activity, and (b) Total ChE activity. AChE and total ChE activity for A4/N1 and A1/N4 groups decreased significantly with increase of the MT concentration (One-way ANOVA, P = 0.0002 for A(a), P< 0.0001 for A(b) and B(b)). The decrease comparison between AChE and total ChE activity from Log [20] concentration = -5 to -1 was analyzed with paired t-test (P = 0.0191 when comparing AChE A(a) with ChE of A4/N1 A(b), P = 0.0046 when comparing AChE B(a) with ChE of A1/N4 B(b)).

AChE activity was measured after tissue constructs were exposed to MT for 20 minutes. AChE activity was reduced significantly from 100% to 70–80% in both A1/N4 and A4/N1 constructs (P = 0.0002 in A4/N1, P< 0.0001 in A1/N4 group, Fig 5). After 20 minutes of MT exposure, BuChE was added to rescue constructs from MT-mediated neurotoxicity. The total ChE activity (AChE and BuChE) decreased significantly with the increase of MT concentration comparing with control in A4/N1 and A1/N4 groups (P<0.0001). From MT concentration 10^−5^ M to 10^−1^ M, total ChE activity decreased significantly compared with AChE activity at both A1/N4 and A4/N1 groups (P = 0.0191 when comparing AChE A(a) with ChE of A4/N1 A(b), P = 0.0046 when comparing AChE B(a) with ChE of A1/N4 B(b)). In the control groups, the group with BuChE (A1/N4: 923±65.1, A4/N1: 990.3±73.6) had higher absorbance compared with the group without BuChE (A1/N4: 416.5±3.1, A4/N1: 445.5±5.3) (Heteroscedastic t-test, P = 0.0059 for A1/N4 group, P = 0.0574 for A4/N1 group).

### *In vitro-in vivo* data comparison

**Fig 6:**
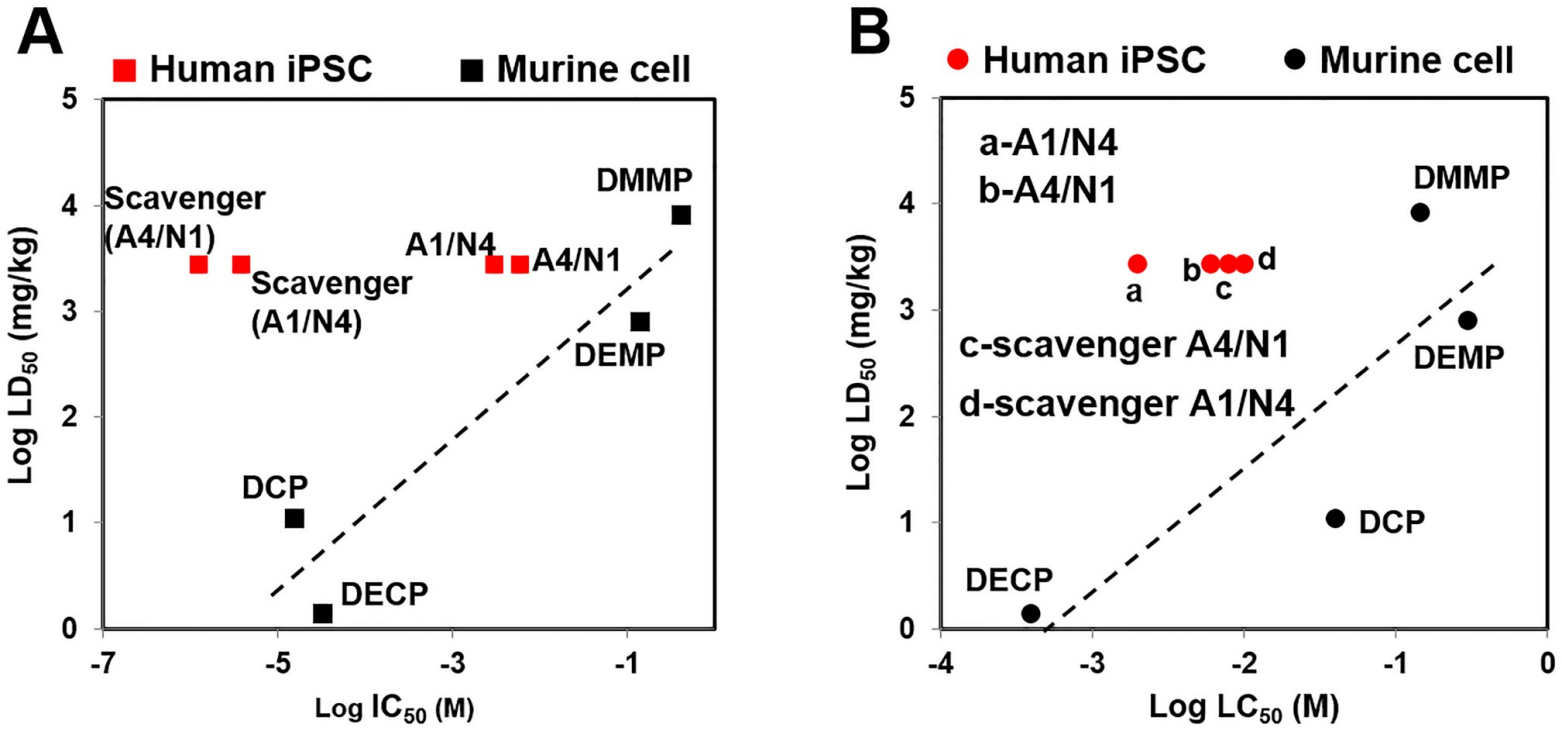
Comparison of *in vitro* ChE activity and viability with *in vivo* LD50 data. A) Log-estimated IC50 for *in vitro* ChE activity vs. LD50 for DMMP, DEMP, DECP, and MT. B) Log-estimated LC50 for *in vitro* viability vs. LD50.

A comparison of the human iPSC-derived brain-on-chip data with *in vivo* toxicity data was added to correlation data from a previous study using the same brain-on-chip model with murine cell lines [21]. ChE activity (inhibitor concentration 50%, IC50), viability (lethal concentration 50%, LC50), and *in vivo* lethal dose 50% (LD50) of previously studied OPs, including MT, are summarized in Table 1. In this study, both groups were exposed to MT with and without BuChE (Scavenger). As shown in Fig 6A, the trend of ChE activity (highest to lowest) was Scavenger-A1/N4 > Scavenger-A4/N1 > A1/N4 > A4/N1. Fig 6B shows the trend of MT-induced toxicity (highest to lowest) was A1/N4 > A4/N1 > Scavenger-A4/N1 > Scavenger-A1/N4. In the absence of BuChE, the A4/N1 group, which had a greater number of astrocytes, showed higher viability. With BuChE, however, the effect was reversed: The A1/N4 group, with a smaller number of astrocytes, showed increased viability instead. In this optimized 3D dynamic brain-on-chip model, we found that BuChE and a higher astrocyte-to-neuron ratio help alleviate the toxicity of MT exposure.

**Table 1:** Comparison of 3D dynamic brain-on-chip co-culture model with *in vivo* data following acute MT exposure.

	*In vivo* (LD50, mg/kg)	ChE activity (IC50, M)	Toxicity (LC50, M)
Dimethyl methylphosphonate (DMMP)	8210	4.113E-01	1.450E-01
Diethyl methylphosphonate (DEMP)	800	1.418E-01	2.950E-01
Diethyl cyanophosphonate (DECP)	1.400	3.229E-05	3.967E-04
Diethyl chlorophosphate (DCP)	11	1.551E-05	3.976E-02
MT-A1/N4	2800	6.000E-03	1.995E-03
MT-A4/N1	2800	3.000E-03	6.026E-03
MT-Scavenger-A1/N4	2800	3.890E-06	1.000E-02
MT-Scavenger-A4/N1	2800	1.259E-06	7.943E-03

## Discussion

We established a brain-on-chip platform using human iPSC-derived neurons and astrocytes mixed in different ratios embedded in 3D matrix. The primary mechanism of acute and chronic neurotoxicity induced by OPs is the inhibition of acetylcholinesterase (AChE) [1]. However, there are also other mechanism of OPs’ toxicity, which are associated with necrosis, apoptosis, and oxidative stress mediated pathway [2]. This paper reports organophosphate-mediated toxicity of 3D brain tissue constructs consisting of human iPSC-derived GABAergic inhibitory neurons (non-cholinergic neurons) and astrocytes, and further explores the potential use of Butyrylcholinesterase (BuChE) as a post-treatment. The 3D tissue construct with GABAergic inhibitory neuron and astrocyte does not directly involved with AChE activity, but MT still induce neurotoxicity to 3D brain tissue.

We found that the cells interacted in 3D networks with synapse formation. And synapses were obviously observed when there were large neuron population in co-culture with astrocytes. Functional synapses have been found not only between neurons, but neurons and astrocytes as well [22], indicating late stage neuronal differentiation [23, 24]. The synapses between astrocytes and neurons also indicate that astrocytes regulate the formation of many types of synapse, including both GABAergic [25, 26] and cholinergic [27, 28]. The signals that astrocytes use to regulate neuronal synapse formation and the pathways activated in neurons in response to these signals are an important area to explore [29]. Furthermore, we found that neurons alone do not grow as well as when co-cultured with astrocytes, which is consistent with previous literature showing that the presence of astrocytes enhances neuronal synaptic activity and accelerates synaptic transmission [30]. These results indicate that the iPSC-derived GABAergic neurons are synaptogenic and require astrocytic support for optimal growth and maturation. The astrocytes effect on viability was also shown in our human iPSC-derived 3D brain-on-chip model [21] (Fig 6). We found that a higher astrocyte-to-neuron ratio improved viability following acute MT exposure. This is consistent with previous study that astrocytes have been found to protect against diazinon-and diazonxon-induced inhibition of neurite outgrowth [31]. In astrocyte-neuron co-culture, astrocytes seem to protect neurons by metabolizing malathion first, leading to astrocyte cell death, which increases the overall cell death in the astrocyte-rich condition, since there are more astrocytes than neurons [32].

Organophosphates are known to inhibit acetylcholinesterase activity in the brain, but it has been reported that this inhibition is not lethal, even at 30~50% inhibition of acetylcholinesterase [33, 34]. There are also other mechanisms of action of Organophosphate. For example, Kashyap et al. reported that lead mitochondrial mediate and caspase regulated apoptosis in brain cells [2, 33]. In a previous study with murine cell lines, the toxicity of various OPs was screened in the same brain-on-chip platform [21]. BMPS was also further validated by screening different types of toxicants and simulated *in silico* physiologically-based pharmacokinetic/pharmacodynamic (PBPK/PD) model, showing good agreement with *in vivo* and *in vitro* data [35]. In this study, we take a step further by using human iPSC-derived cells to mimic human brain structure and function. Human iPSC-derived cells carry proliferation and differentiation properties that are closer to the human *in vivo* situation and have been widely applied in drug screening and diseases models [36, 37]. The 3D cell assembly properties of iPSCs are able to recapitulate endogenous biological systems [38], which 2D cell culture, transwell technology and 3D cell-line culture could not recapitulate. Current 2D *in vitro* models do not recapitulate the continuous interaction of multiple tissues, nor predict brain physiology. Similarly, brain slices may provide a certain level of brain circuit function, but cannot simulate the complex, multifunctional, and integrated neuro-glial system needed to explore neurotoxicity and screen compounds for better treatment. Animal models are also limited, in that (i) they do not replicate the full spectrum of human neuropathology and associated functional deficits, and (ii) they are complicated by ethical issues. Eventually this platform can provide clinically relevant information for a better understanding of toxicity mechanisms and for diagnostics, personalized medicine, drug screening, and to possibly reduce the need for animal models. We co-cultured different ratios of human iPSC-derived neurons and astrocytes to model the human brain in an *in vitro* 3D platform.

Co-cultured neurons and astrocytes were exposed to MT and then treated with BuChE to assess its function as a bioscavenger of OPs. One of the known mechanisms of OP toxicity is AChE inhibition, subsequent ACh accumulation in synapses and neuromuscular junctions, and induction of hyper-cholinergic activity [39–43]. Exogenously administered bioscavengers, such as BuChE counteract OP exposure by binding and sequestering OPs, preventing inhibition of AChE [44]. BuChE is pseudocholinesterase, a nonspecific cholinesterase enzyme that hydrolyses various choline-based esters. Both AChE and BuChE are able to convert the acetylcholine substrate to choline, which data are shown in Fig 5.

Since the amount of BuChE added was much greater than the amount of cell-derived AChE, the decrease in total ChE was mostly due to BuChE binding and sequestering MT. This explains the significant drop of total ChE (AChE + BuChE) compared to AChE alone in the presence of MT (Fig 5). Different from AChE, BuChE hydrolyzes butyrylcholine more quickly than ACh [45]. The toxicity of OPs results from irreversible inhibition of AChE and the subsequent continuous stimulation of neurons by ACh [46]. Exogenous BChE binds OPs irreversibly to occupy the binding points of OPs for AChE, preventing inactivation of AChE and continuous cholinergic stimulation [47]. Administration of BuChE as a scavenger is a potential strategy for preventing toxicity from OP agents. When comparing this result to previous correlation data [21], we found that adding BuChE to MT exposed coculture decreased ChE activity and increased viability (Fig 6). Furthermore, the addition of BuChE seemed to reverse the effect of a higher astrocyte-to-neuron ratio regarding improved viability, indicating that bioscavengers are more effective at alleviating OP toxicity than astrocyte-mediated mechanisms. Overall, these results suggest that we successfully established a model for OP toxicity with co-cultured human iPSC-derived cells, and developed countermeasure screening using the human brain-on-chip platform.

## Supporting information

S1 Fig Bright-field images of human iPSC-derived (A) astrocytes and (B) GABAergic neurons in 2D culture. (TIF)
