# Supplementary figures and images for: Three-dimensional brain-on-chip model using human iPSC-derived GABAergic neurons and astrocytes: Butyrylcholinesterase post-treatment for acute malathion exposure
Source: PLoS One. 2020 Mar 12;15(3):e0230335. doi: 10.1371/journal.pone.0230335 (PMC7067464; doi:10.1371/journal.pone.0230335)

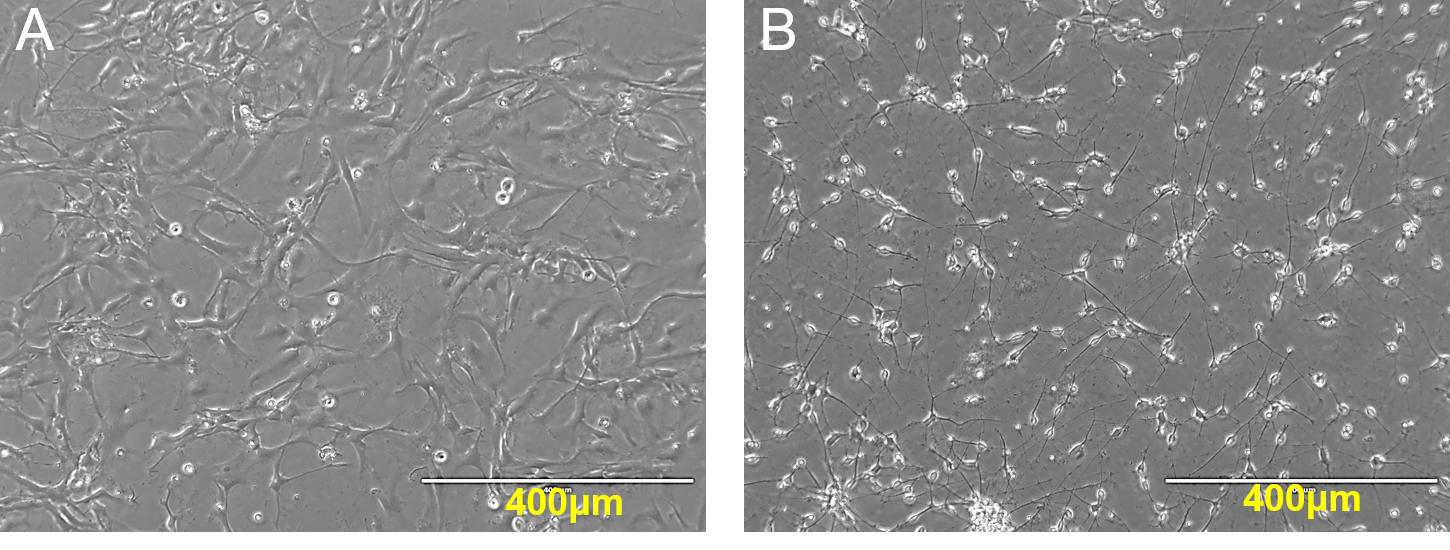

Supplement: S1 Fig — (TIF) [file pone.0230335.s001.tif]
